# Effect of C-reactive protein on the risk of Heart failure: a mendelian randomization study

**DOI:** 10.1186/s12872-023-03149-3

**Published:** 2023-03-07

**Authors:** Danial Habibi, Maryam S Daneshpour, Sara Asgarian, Karim Kohansal, Farzad Hadaegh, Marjan Mansourian, Mahdi Akbarzadeh

**Affiliations:** 1 Department of Biostatistics and Epidemiology, School of Health, and Student Research Committee, School of Health, Isfahan University of Medical Sciences, Isfahan, Iran; 2 Cellular and Molecular Endocrine Research Center, Research Institute for Endocrine Sciences, Shahid Beheshti University of Medical Sciences, Tehran, Iran; 3 Prevention of Metabolic Disorders Research Center, Research Institute for Endocrine Sciences, Shahid Beheshti University of Medical Sciences, Tehran, Iran; 4 Epidemiology and Biostatistics Department, School of Health, Isfahan University of Medical Sciences, Isfahan, Iran; 5 Cellular and Molecular Endocrine Research Center, Research Institute for Endocrine Science, Shahid Beheshti University of Medical Science, P.O. Box: 19395-4763, 1985717413 Tehran, Iran

**Keywords:** C-reactive protein, Heart failure, Two-sample mendelian randomization, Interleukin

## Abstract

**Background:**

Traditional observational studies have shown positive associations between c-reactive protein (CRP) and heart failure (HF) risk. However, this association has not been fully elucidated. Therefore, Mendelian randomization was used to examine CRP’s possible etiological roles with HF.

**Methods:**

We implemented a two-sample Mendelian randomization framework to examine the causality of the association between CRP and HF based on summary statistics by large-scale genome-wide association studies (GWAS) datasets of European ancestry through inverse-variance weighted, weighted median, MREgger regression, and MR-PRESSO methods. The summary statistics dataset on the association of genetic variants with CRP was used from the published GWAS of European descent in UK Biobank participants (N = 427,367) and the CHARGE consortium (N = 575,531). The GWAS dataset used to identify genetic variants underlying HF from the HERMES consortium includes 977,323 participants (47,309 cases and 930,014 controls). The odds ratio (OR) with 95% confidence intervals (CIs) was employed to examine this association.

**Results:**

The results of our IVW indicated that CRP was strongly associated with HF (OR = 4.18, 95% CI = 3.40–5.13, p < 0.001). The Cochran heterogeneity test showed significant heterogeneity among SNPs of CRP (Q = 317.55, p < 0.001; I^2^ = 37.6%), and no considerable pleiotropy was detected for the association of CRP with HF [intercept = 0.003; p = 0.234]. This finding remained consistent using different Mendelian randomization methods and sensitivity analyses.

**Conclusion:**

Our MR study did identify convincing evidence to support CRP associated with HF risk. Human genetic data suggest that CRP is a causative factor in HF. Hence, CRP assessment may offer additional prognostic information as an adjuvant to overall risk assessment in HF patients. These findings prompt significant questions about the function of inflammation in the progression of HF. More research into the role of inflammation in HF is needed to guide trials of anti-inflammation management.

**Supplementary Information:**

The online version contains supplementary material available at 10.1186/s12872-023-03149-3.

## Introduction

Heart failure (HF) as a heterogeneous syndrome has been the subject of ongoing interest since it was first described as an emerging epidemic about 25 years ago [1]. It has been estimated that 64.3 million people suffer from HF worldwide. The prevalence will rise due to improved HF patients’ survival and generally increased life expectancy in the general population [2]. HF is the leading cause of hospitalization in patients over 65, accounting for 1–2% of all hospitalizations in the Western world, and its high burden is associated with substantial expenditures on patients and public health systems [3].

Systemic inflammation with elevated circulating pro-inflammatory biomarkers levels in acute and chronic HF patients is a common pathobiological condition that has opened a new area of research about the possibly important role of the immune system in the pathogenesis of HF. The TIME-CHF (Trial of Intensified vs. Standard Medical Therapy in Elderly Patients With Congestive Heart Failure) trial reported high circulating levels of high-sensitivity C-reactive protein (hs-CRP) in patients with stable chronically reduced ejection fraction and preserved ejection fraction (EF) heart failure [4]. Considering acute HF, in the ASCEND-HF (Acute Study of Clinical Effectiveness of Nesiritide in Decompensated Heart Failure) trial, systemic inflammation and elevated hs-CRP levels were also observed in patients [5].

Interleukin 1 (IL-1), a cytokine that acts as a master regulator of inflammation, triggers a cascade of inflammatory mediators by activating the IL-1 receptor, leading to the stimulation of IL-6 secretion [6]. Interleukin (IL)-1 signalling leads to impaired systolic and diastolic function through various mechanistic pathways and may play a pivotal role in the pathogenesis of HF [7]. Therefore, various clinical trials have been conducted on anti-inflammatory therapies for patients with HF. In 2019, the Canakinumab Anti-Inflammatory Thrombosis Outcomes Study (CANTOS) findings showed that anti-cytokine therapy with a monoclonal antibody against IL-1β improved heart failure outcomes in patients with myocardial infarction with or without established heart failure [8].

Utilizing genetic variants as instrumental variables for an exposure, Mendelian randomization (MR) can improve the causal inference of an exposure-outcome association. It minimizes potential methodological limitations, such as confounding and reverse causality [9]. Here, we aimed to perform a two-sample MR analysis to test the hypothesis CRP on HF risk in a European population.

## Method

### Selection of genetic variants

**Fig. 1:**
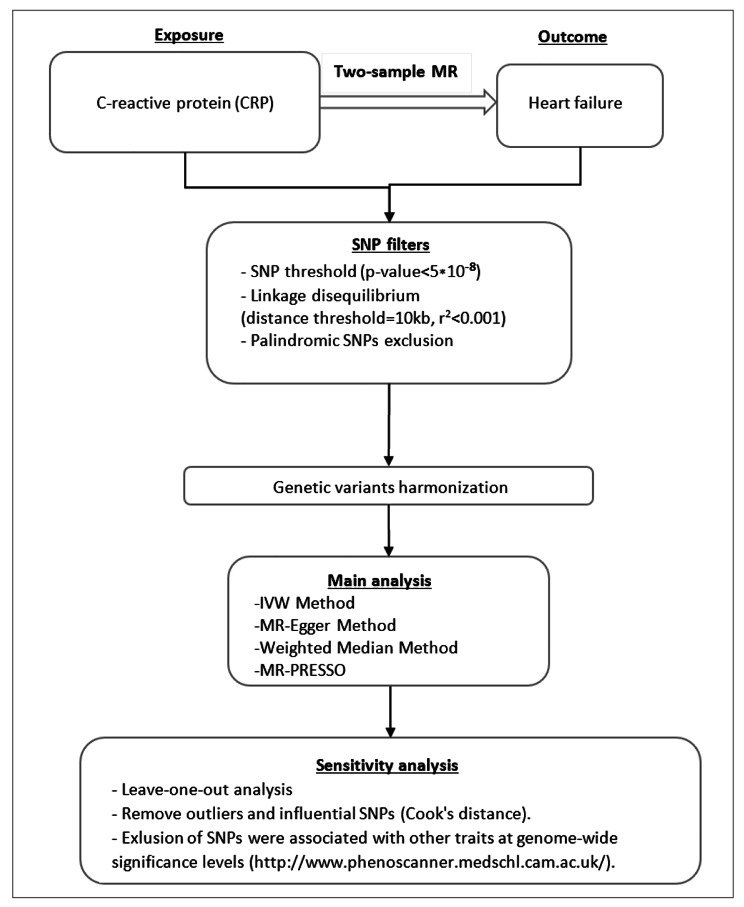
The steps of Mendelian randomization (MR) analysis

We used summary genetic information of the European ancestry on genetic variants associated with CRP from Dehghan et al. [10]. GWAS summary data on heart failure were obtained from Shah et al. [11]. In this step, SNPs need to be ensured as valid instrument variables (IVs): (i) SNPs are strongly associated with exposure and significant according to p < 5.0 × 10^ − 8^; (ii) SNPs are independent of each other to avoid biases caused by linkage disequilibrium (r^2^ < 0.001 over a 10-kilobase (kb) region based on the European sample of 1000 Genomes data), and (iii) SNPs could impact other than exposure on the outcome (Additional file 1: Figure S1). To assess whether the selected SNPs were associated with other traits at genome-wide significance levels, we applied the PhenoScanner (http://www.phenoscanner.medschl.cam.ac.uk/). We then assessed the results after excluding these pleiotropic SNPs. The relevant information was extracted, including chromosome, gene, effect allele, non-effect allele, effect allele frequency, effect sizes, standard error, and P-value. To avoid weak instrumental bias, the F-statistic of the selected SNPs was calculated to test the weak IV bias for our MR study. If the F-statistics > 10, the strength of selected single-IVs was strong [12]. A step-by-step workflow in this study is presented in Fig. 1.

### Statistical analysis

The inverse variance-weighted (IVW) method was performed to primarily analyze the causal associations between exposures (CRP) and outcome (HF) [13]. Moreover, we applied several other approaches, including weighted median, MR-Egger, and MR-PRESSO, Maximum likelihood method and Robust Adjusted Profile Score (RAPS) to detect the robustness of our results [14]. We further executed a leave-one-out sensitivity analysis to assess whether individual SNPs influenced the results.

For detecting heterogeneity and pleiotropy, we used Cochran’s Q statistic and the I^2^ index for MR-inverse-variance weighted analyses and Rucker’s Q statistic for MR-Egger [15]. Besides, we used the MR-Egger method (by intercept tests) to evaluate horizontal pleiotropy [16]. We further used Cook’s distance and Studentized residuals to ascertain whether any individual SNPs were detected as outliers and influential points in driving the analysis results [16]. To test the direction of causations, we confirmed by employing the MR Steiger test to determine whether the observed associations were directionally causal, P < 0.05 was defined as statistically significant [17]. All statistical analysis was calculated with the help of R software (version 4.0.3) by “TwoSampleMR”, “MendelianRandomization”, “MR-PRESSO” and " mr.raps” packages [18–20].

## Results

**Fig. 2:**
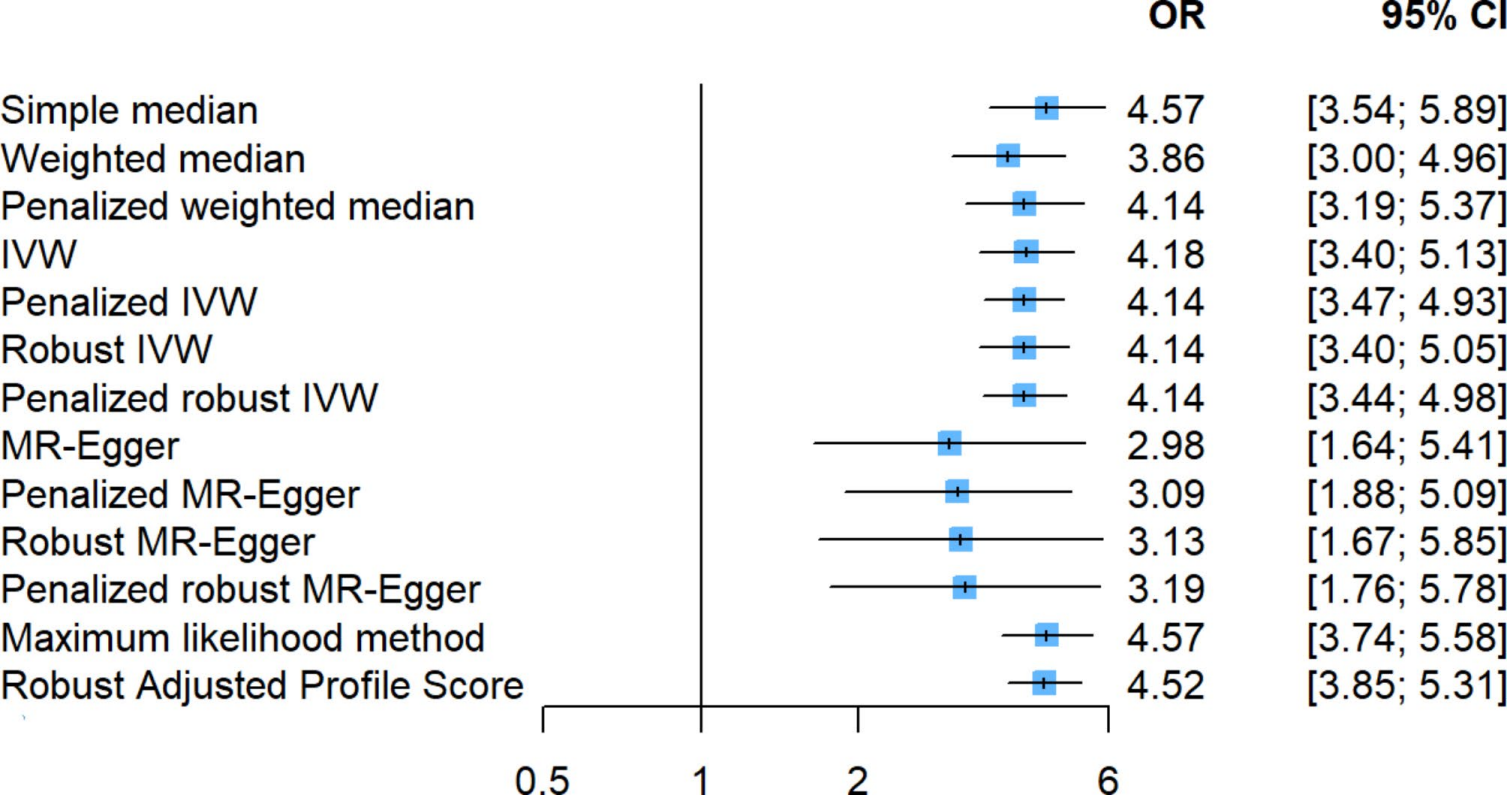
Comparison of the causal estimates from the various Mendelian randomization methods

We assessed the potential relationship between HF and CRP. There was clear evidence of a causal effect of CRP on HF in Table 1 (P < 0.05 in the three MR methods). The Cochran heterogeneity test showed significant heterogeneity among SNPs of CRP (Q = 426.42, p < 0.001; I^2^ = 48.4%), and no significant pleiotropy was detected for the association of CRP with HF [intercept = 0.002; p = 0.530]. The IVW analysis showed that CRP was associated with an increased risk of developing HF (OR = 4.37, 95% CI = 3.58–5.34, p = < 0.001). The causal inference of the sensitivity analysis conducted using the weighted median method (OR = 3.86, 95% CI = 2.98–5.01, p = < 0.001). After excluding 3 SNPs (rs12567136, rs12740374 and rs9295128) with potential outliers and influential points, as well as, potential pleiotropy (11 SNPs), significant causal effect between CRP and HF was observed (Q = 317.55, p < 0.001; I^2^ = 37.6%; intercept = 0.003, p = 0.234; IVW, OR = 4.18, 95% CI = 3.40–5.13, p < 0.001) (Table 1, Additional file 1: Figure S2 and Figure S4). The results of the causal association between CRP and HF is depicted in Fig. 2 (Additional file 1: Figure S6). The causal assumption of CRP and HF was verified via the MR Steiger test, and the result showed CRP’s influence on HF was the correct causal direction (P = < 0.001).

**Table 1:** Mendelian randomization (MR) estimates of CRP with HF

*Methods*	*IVs (SNPs)*	*OR* ^*a*^ *(95% CI)*	*P-value*	*Q-statistics*	*Ph*
*IVW*	*200*	*4.18 (3.40, 5.13)*	*< 0.001*	*317.55*	*< 0.001*
***WM***	***200***	***3.86 (3.01, 4.97)***	***< 0.001***		
*MR-Egger*	*200*	*2.98 (1.64, 5.40)*	*< 0.001*		
*MR-PRESSO*	*195*	*4.18 (3.59, 5.36)*	*< 0.001*		
*MR-Egger intercept* ^*b*^	*200*	*0.003 (-0.002, 0.008)*	*0.234*		

The best causal estimation is highlighted in bold

IVW: Inverse variance weighted; WM: Weighted median; CI: confidence interval; Ph: Heterogeity p-value

^a^Odds ratio per 1 SD increase.

^b^Regression coefficient (95% CI).

Moreover, we assessed the potential relationship between HF and CRP. During the process of harmonization, two SNPs (rs1556516 and rs4766578) were removed for being palindromic. Finally, 10 SNPs were selected as genetic instruments for analyzing (Additional file 1: Figure S8). The Cochran heterogeneity test showed significant heterogeneity among SNPs of HF (Q = 216.34, p < 0.001; I^2^ = 95.8%), and no significant pleiotropy was detected for the association of HF with CRP [intercept = 0.0001; p = 0.984]. The IVW analysis showed that HF was associated with an approximately 8% increased risk of developing CRP (OR = 1.08, 95% CI = 1.03–1.14, p = 0.004). The causal inference of the sensitivity analysis conducted using the weighted median method (OR = 1.03, 95% CI = 1.01–1.06, p = 0.007), the MR-PRESSO method (OR = 1.06, 95% CI = 1.03–1.09, p = 0.042), and RAPS method (OR = 1.12, 95% CI = 1.10–1.14, p = < 0.001).

### Sensitivity analysis

We chose the MR-Egger method to evaluate the potential pleiotropy effects. We also applied the MR pleiotropy residual sum and outlier test (MR-PRESSO) methods and MR pleiotropy residual sum and outlier (Radial MR) to assess the presence of and the outlying SNPs, and reassess the effect estimates.

To identify the potential outliers and influential points, we used Cook’s distance and Studentized residuals (Additional file 1: Figure S3). We also performed the leave-one-out analysis to test the influence of outlying values (Additional file 1: Figure S5). We further explored the pleiotropy of each selected SNPs to remove the effect of other confounders through the PhenoScanner. Moreover, we depicted funnel plot of causal association for CRP (Additional file 1: Figure S7) on heart failure. Weak instrumental variable was assessed through the F-statistic (Additional file 2: Dataset). We also checked SNPs within the HLA region that did not exist in our MR analysis.

## Discussion

Using two-sample Mendelian randomization, we found that genetically determined CRP was causally associated with the risk of heart failure in the European population. The aim of this study was to evaluate a potentially causal relationship between CRP and heart failure, and the Mendelian randomization analysis was chosen because it mitigates the limitations of observational studies such as reverse causation and unmeasured confounding. Our results suggested a significant positive association between CRP and HF, with causal estimation ranging from 2.98 to 4.57 OR in various Mendelian randomization methods Alternatively, a 1 unit increase in genetically predicted CRP levels was linked to an increased risk of heart failure.Sensitivity analyses were conducted to ensure the accuracy of the findings.

In recent years, inflammation has attracted attention as a key pathophysiological factor in heart failure syndrome and as a potential factor in heart failure progression, followed by multiple biomarkers reflecting the activation of inflammatory pathways that have been characterized as outcome predictors [21]. CRP, as the first known inflammatory biomarker to be elevated in patients with heart failure, was proposed in 1956 [22]. In the following, various studies investigated the prognostic values of elevated CRP levels with the incidence and features of more severe heart failure; several large studies have shown that CRP predicts morbidity and mortality in patients with established HF [23–27]. In the Valsartan Heart Failure Trial (Val-HeFT), CRP was an independent predictor of mortality and morbidity in patients with established HF [24]. Evidence from observational cohort studies suggests that elevated CRP is a risk factor for the incidence of heart failure independent of conventional risk factors and other biomarkers [28–30], indicating that these inflammation biomarkers may play etiological roles in HF.

Additionally, prior studies have shown a strong relationship between elevated CRP levels and the hospitalization of HF patients, regardless of the severity of CHD, medication use, baseline heart failure, or subsequent MI events [31, 32]. Based on these findings, a greater degree of CHD does not play a role in mediating the link between CRP and HF [31]. Nevertheless, in 2019, a meta-analysis with 9,289 established heart failure patients reported that hs-CRP did not provide more prognostic information than strong outcome predictors such as NT-proBNP and high-sensitivity troponin T (hs-TnT) [33]. Although traditional epidemiological studies have revealed a link between CRP and the risk of heart failure, presumably supporting the hypothesis that there is a cause and effect connection, they may include limitations and various biases. This is, to our knowledge, the first research of the association between CRP and HF on European population with GWAS data sources for both exposure and outcome using MR approaches. A recent MR study on East Asian people found suggestive evidence of a causal relationship between CRP and congestive heart failure in their IVW approach, which is consistent with the findings of our analysis, though conducted on a different community [34]. Another MR research on European populations in 2021 assesses the causative effects of 27 risk factors on the incidence and mortality of heart failure and reveals suggestive connections between genetically predicted CRP and heart failure incidence. Nevertheless, following Bonferroni correction, none of their modifiable risk variables were shown to be significantly associated to the incidence rate or mortality of heart failure [35]. When compared to this study, we used the largest genome-wide association study (GWAS) on CRP as our exposure database, which made our results more extendable, trustworthy, and potent. Another MR study exploring the link between inflammatory markers and heart failure (HF) has yielded a different result, concluding that genetically predicted CRP was not likely to cause HF and that there was only weak evidence of a causal effect between the two [36]. Another study by Remmelzwaal et al. in 2022 used GWAS in a population of European ancestry to investigate the causal relationship between various inflammatory biomarkers and the risk of new-onset heart failure [37]. Contrary to our findings, they were unable to establish a causal relationship between CRP and heart failure risk. It is worth noting that the HF summary data from GWAS differed between our study and the mentioned one. Furthermore, as a limitation discussed in the study by Remmelzwaal et al., weak instrument bias may also play a role in this dimorphism between studies.

Several mechanisms have been considered to elucidate the association between inflammatory biomarkers and HF. Interleukin-6 (IL-6) is a strong inducer of CRP manufacture in hepatocytes and is generated by several cell types, including cardiomyocytes, in response to hypoxic stress [38]. Left ventricular dysfunction, low cardiac output, hypo perfusion, and venous congestion have all been indicated as possible factors for IL-6 and CRP secretion. IL-6 has been demostrated to be enhanced in diseases with asymptomatic systolic dysfunction [39], and enhanced IL-6 and CRP levels have been illustrated to predict HF events in an asymptomatic elderly population [40]. The cytokine supposition of HF postulates that the progression of HF is motivated by the activation of inflammatory intermediates and their adverse effects on the cardiovascular system. Therefore, in the Heart and Soul study, the follow-up of patients with established CHD indicated that heart failure incidence emerges to be relatively elucidated by abnormal diastolic function in patients with high CRP levels [31].

Compared to previous studies, our results are based on recent data, and we were able to find more genes associated with HF. Briefly, our analysis offers evidence that CRP, a risk factor, plays a significant role in the development of heart failure. This highlights the importance of strategies for primary prevention, early detection, and managing heart failure, which will serve as valuable benchmarks for improving patient prognosis. Additionally, preventative cardiovascular services should be made available to underprivileged communities to ensure fair access to healthcare.

A major strength of our study was the design of the MR analysis, which minimizes biases from residual confounding and reverse causation. Additionally, data from large GWAS of risk factors were used to lessen the confounding effect of population stratification, and only cohorts with a predominance of European ancestry were allowed to provide data. Our study was conducted under the confirmation of the MR study’s three critical assumptions. The results were robust and supported by various MR approaches, including IVW, weighted median, MR-Egger and MR-PRESSO. We also conducted additional analyses that excluded SNPs with potential influence, outliers, and pleiotropy, which can reduce the bias in estimations of the causal effect. The leave-one-out sensitivity analysis indicated that the overall effect was not driven by a single SNP, demonstrating the consistency and accuracy of our results. Meanwhile, several of the mechanisms mentioned previously lend credence to the biological plausibility of our findings.

Nevertheless, our study has several limitations. First, the data on subtypes of disease outcomes were unavailable, thus we could not conduct an analysis stratified by subtypes and severity of HF. Second, we only evaluated the causal relationship between certain inflammatory biomarkers and HF. Hence, our results should be treated with caution, and we also need to further evaluate the etiological roles of other inflammatory factors in HF. Third, sensitivity analyses was not be performed using data from patients with HFpEF versus HFrEF.

## Conclusion

In summary, our findings provide genetic support for a potential causal relationship between c-reactive protein and heart failure. Elevated c-reactive protein levels were associated with an increased risk of heart failure. Focusing on the guiding role of c-reactive protein (CRP) could function in the future prevention and treatment of heart failure.

## Electronic supplementary material

Below is the link to the electronic supplementary material.

Additional file 1: Mendelian randomisation analysis estimates from GWAS results from HF and selected phenotypes obtained with CRP.

Additional File 2: Description of supplementary materials

## Data Availability

The original data used are publicly available at https://gwas.mrcieu.ac.uk/ and https://www.ebi.ac.uk/gwas/. The original contributions presented in the study are included in the article/Supplementary Material; further inquiries can be directed to the corresponding author.

## Abbreviations

CRP: C-reactive protein
HF: Heart failure
IVW: Inverse variance weighted
WM: Weighted median
GWAS: Genome-wide association study
IV: Instrumental variable
MR: Mendelian randomization
