# Supplementary material for: Effect of C-reactive protein on the risk of Heart failure: a mendelian randomization study
Source: BMC Cardiovasc Disord. 2023 Mar 7;23:112. doi: 10.1186/s12872-023-03149-3 (PMC9993577; doi:10.1186/s12872-023-03149-3)

Supplementary materials

**Effect of** **C-reactive Protein on the Risk of Heart failure: A Mendelian Randomization Study**

1. Danial Habibi, Ph.D. student, Biostatistics, Department of Biostatistics and Epidemiology, School of Health, Student Research Committee, Isfahan University of Medical Sciences, Isfahan, Iran. Email:[dhabibi67@gmail.com](mailto:dhabibi67@gmail.com)
2. Maryam S Daneshpour, Ph.D., Cellular and Molecular Research Center, Research Institute for Endocrine Sciences, Shahid Beheshti University of Medical Sciences, Tehran, Iran. Email:[daneshpour@sbmu.ac.ir](mailto:daneshpour@sbmu.ac.ir)
3. Sara Asgarian, M.D., Cellular and Molecular Research Center, Research Institute for Endocrine Sciences, Shahid Beheshti University of Medical Sciences, Tehran, Iran. Email:[sara.asgn@gmail.com](mailto:sara.asgn@gmail.com)
4. Karim Kohansal, M.D., Prevention of Metabolic Disorders Research Center, Research Institute for Endocrine Sciences, Shahid Beheshti University of Medical Sciences, Tehran, Iran. Email:[karimkohansal@sbmu.ac.ir](mailto:karimkohansal@sbmu.ac.ir)
5. Farzad Hadaegh, M.D., Prevention of Metabolic Disorders Research Center, Research Institute for Endocrine Sciences, Shahid Beheshti University of Medical Sciences, Tehran, Iran. Email:[fzhadaegh@endocrine.ac.ir](mailto:fzhadaegh@endocrine.ac.ir)
6. Marjan Mansourian, Ph.D., Epidemiology and Biostatistics Department, School of Health, Isfahan University of Medical Sciences, Isfahan, Iran.

Email: [jmansourian@gmail.com](mailto:jmansourian@gmail.com)

1. Mahdi Akbarzadeh, Ph.D., Biostatistics, Cellular and Molecular Research Center, Research Institute for Endocrine Sciences, Shahid Beheshti University of Medical Sciences, Tehran, Iran. Email: [akbarzadeh.ms@gmail.com](mailto:akbarzadeh.ms@gmail.com)

****Correspond authors:***

*First: Marjan Mansourian.*

Epidemiology and Biostatistics Department, School of Health, Isfahan University of Medical Sciences, Isfahan, Iran. Email: [jmansourian@gmail.com](mailto:jmansourian@gmail.com)

*Second: Mahdi Akbarzadeh.*

Cellular and Molecular Endocrine Research Center, Research Institute for Endocrine Science, Shahid Beheshti University of Medical Science, Tehran, Iran. Email: [akbarzadeh.ms@gmail.com](mailto:akbarzadeh.ms@gmail.com),

P.O. Box: 19395-4763, 1985717413,

Tel: +98 (21) 22432500, Fax: +98 (21) 22402463

Table Contents

[Figure S1. Diagram of Mendelian randomization framework in the current paper.](#_Toc29464912) 3

Figure S2. A step-by-step workflow for c-reactive protein ………………………………………………………………...4

[Figure S3. The result of Cook’s distance and Studentized residuals for c-reactive protein .](#_Toc29464922) 5

[Figure S4. Comparison of the causal estimates from the various Mendelian randomization methods and scatter plot of the potential effects of CRP-association SNPs on HF..](#_Toc29464935) 7

[Figure S5. Leave-one-out plot to assess if a single variant is driving the association between c-reactive protein and heart failure](#_Toc29464936) 9

[Figure S6. Forest plot of variant specific inverse variance estimates for causal association between c-reactive protein and heart failure..](#_Toc29464937) 10

[Figure S7. Funnel plot of causal association between c-reactive protein and heart failure.](#_Toc29464938) 11

[Figure S8. Causal association between heart failure and c-reactive protein .](#_Toc29464938) 12

**Figure S1. Diagram of Mendelian randomization framework in the current paper.**

MR utilizes genetic variation as an instrumental variable (IV) to investigate the causal association between exposure and outcome in non-experimental data (1). To utilize a genetic variant to be a valid instrumental variable, several main assumptions should be satisfied. The IVs (SNPs) are strongly associated with exposure (s) and should be clear quantifiably. (ii) The SNPs are not linked with any confounder of the exposure-outcome association. (iii) The SNPs do not affect the outcome, except possibly through their association with the exposure (s).


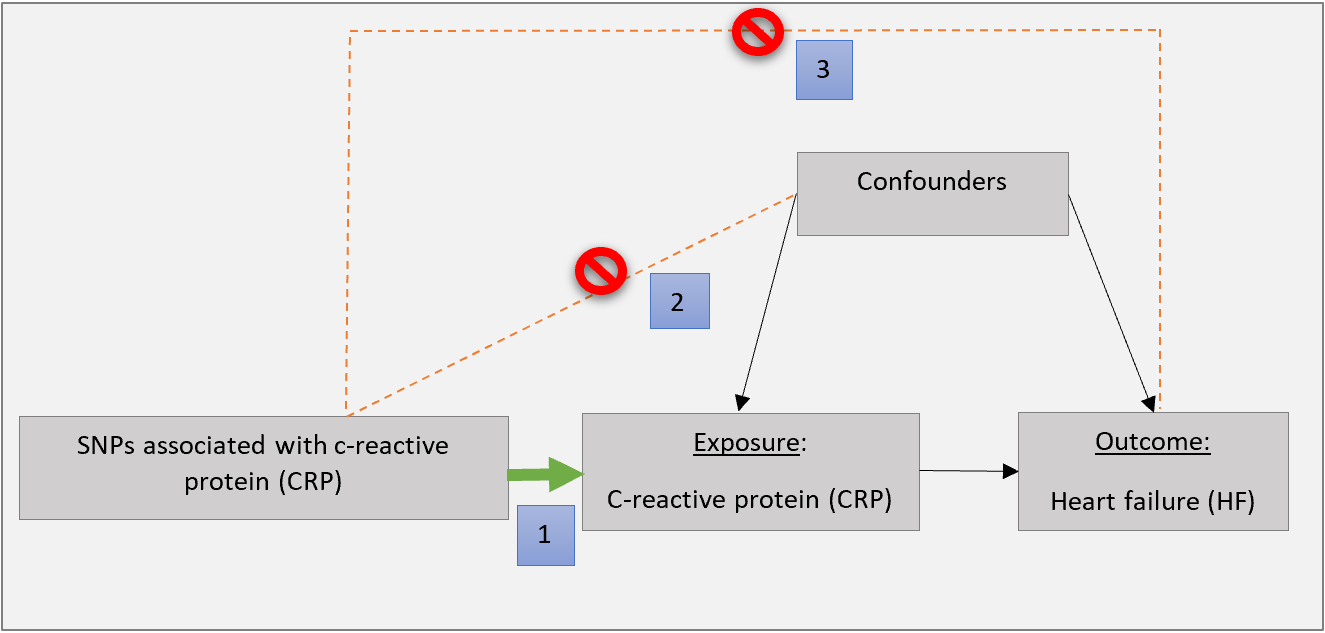


The IV techniques are a few available ways to estimate the causal effects without the full knowledge of all the confounders of the exposure-outcome association. The IV is referred to as an external variable (SNPs) that is associated with exposure. Besides, it is independent of the outcome and any factor linked to it, other than exposure.

**Figure S2. A step-by-step workflow for c-reactive protein (CRP).**

GWAS summary data of CRP

SNPs associated with CRP

Selected SNPs related to p-value<5.0×10^-8^

and removing SNPs associated with LD clumping

SNPs both in CRP and HF

Removing ambiguous and palindromic SNPs of which the effect cannot be corrected in harmonizing (Action=2) process. Moreover, remove the potential outliers and influential points (Cook's distance and Studentized residuals) and weak instrumental SNPs (F-statistics<10)

Exclusion SNPs related to other traits

(<http://www.phenoscanner.medschl.cam.ac.uk/>)

Instrumental SNPs

IVW method

Weighted median

MR-egger method

Heterogeneity

No

Horizontal pleiotropy

No

Yes

Yes

Draw a conclusion with caution

Draw a conclusion with positive attitude

Is sensitivity analysis significant?

**Figure S3. The result of Cook’s distance and Studentized residuals for c-reactive protein.**


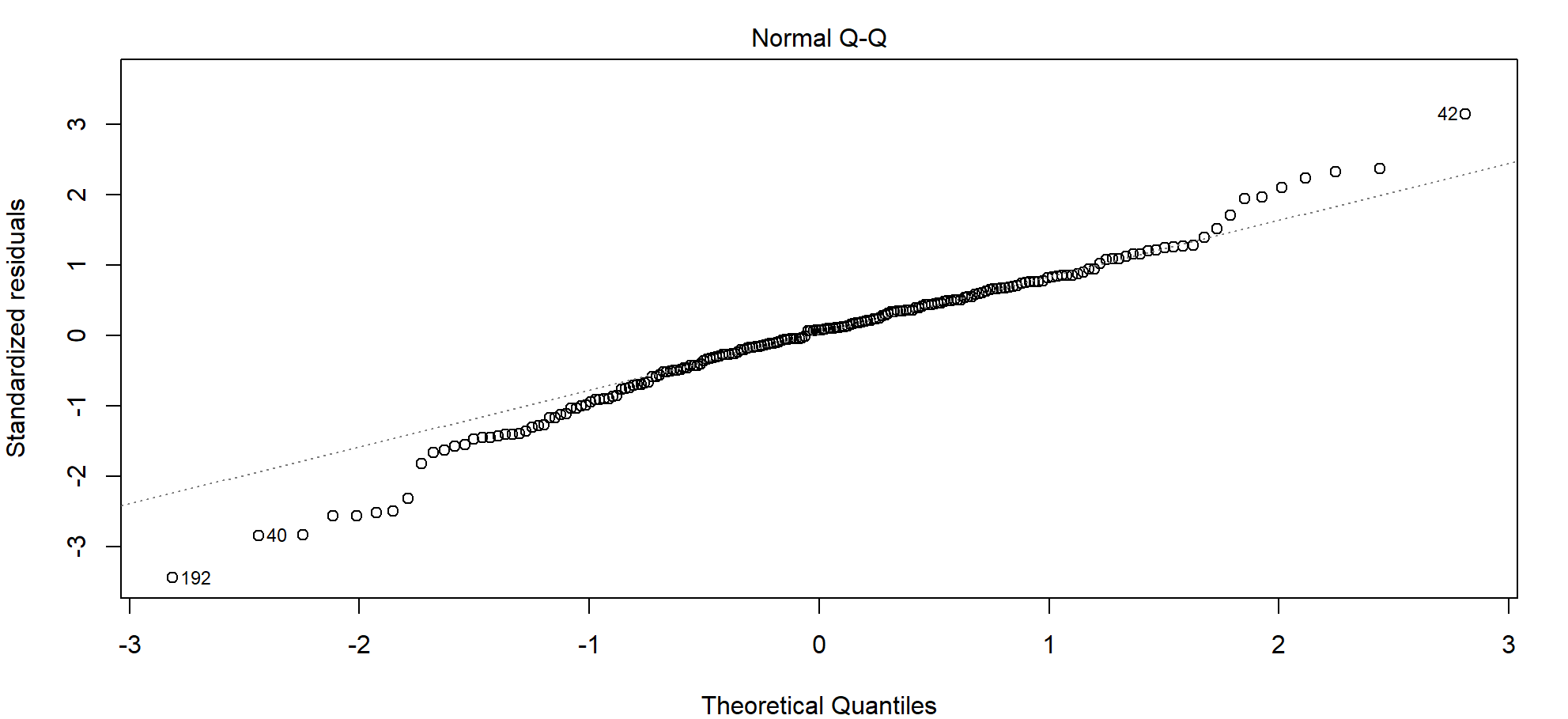


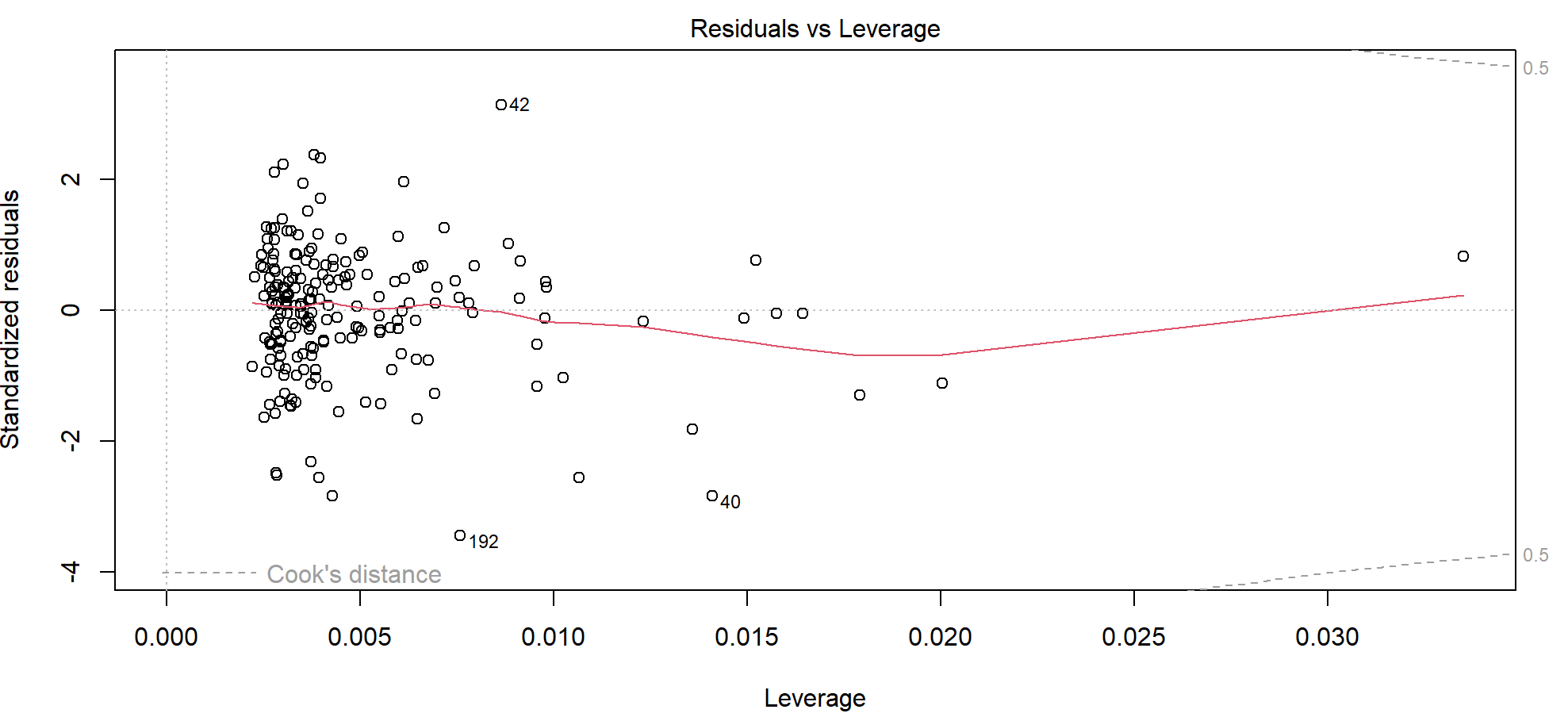


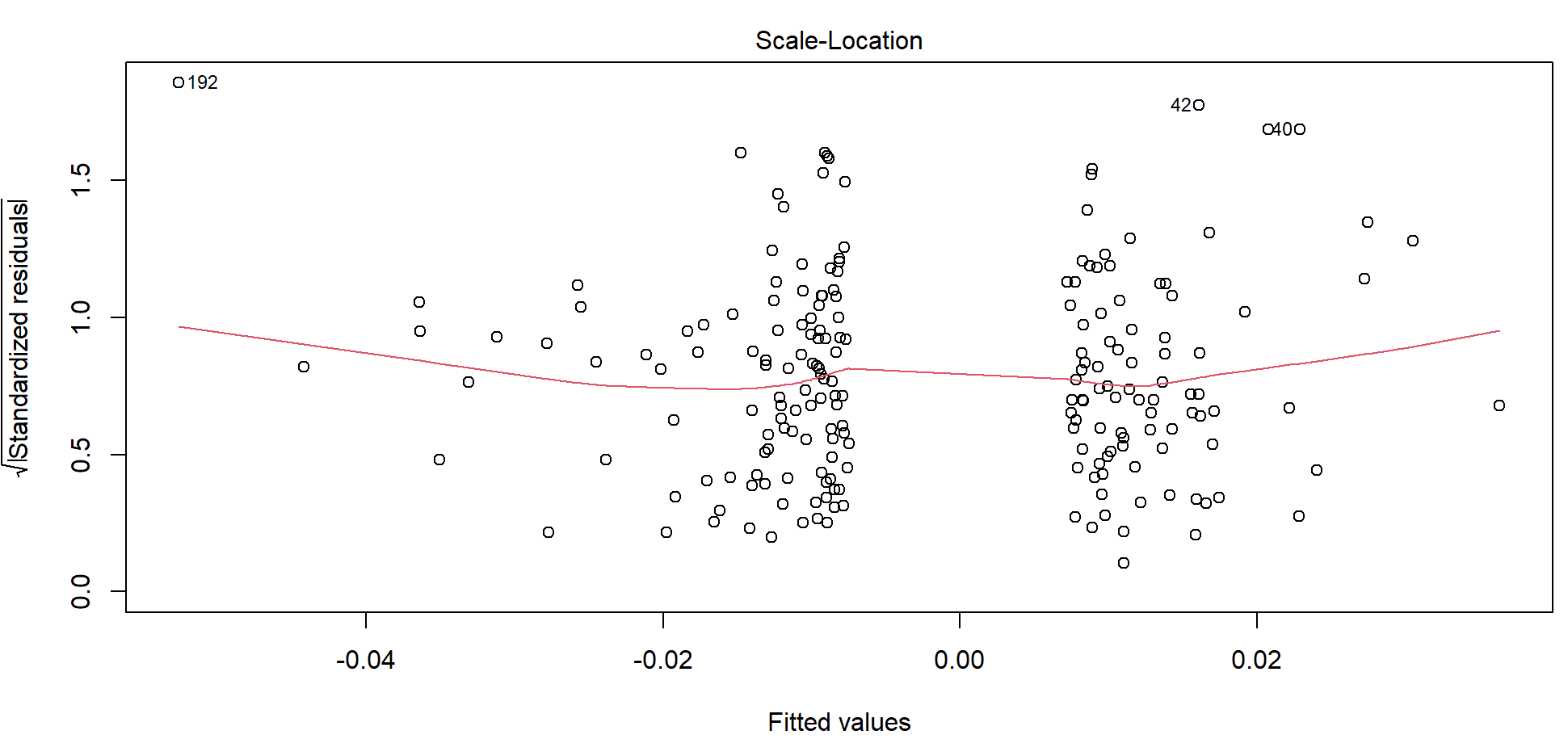


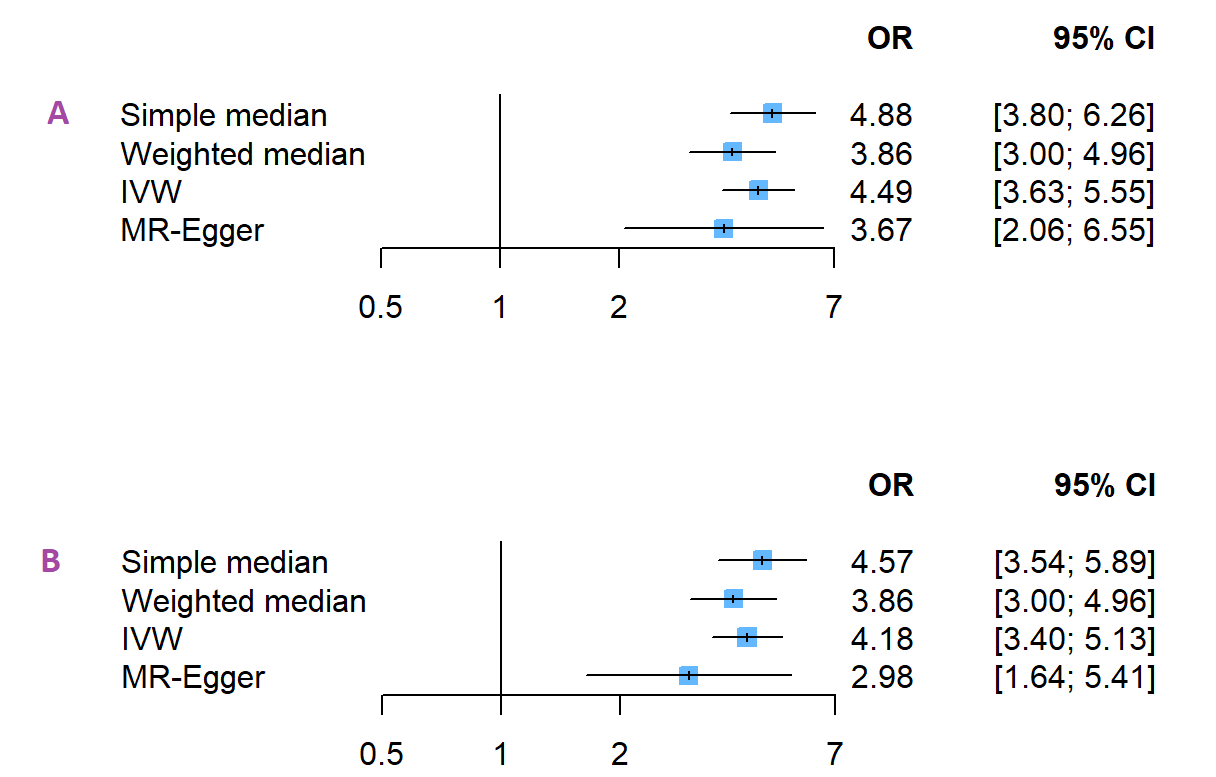
 A: Main analysis (with potential outliers, influential points and pleiotropy); B: After used Cook's distance (remove outliers); IVW: Inverse variance weighted; OR: odds ratio; CI: confidence interval.

**Figure S4: Scatter plot of the potential effects of CRP-association SNPs on heart failure.**


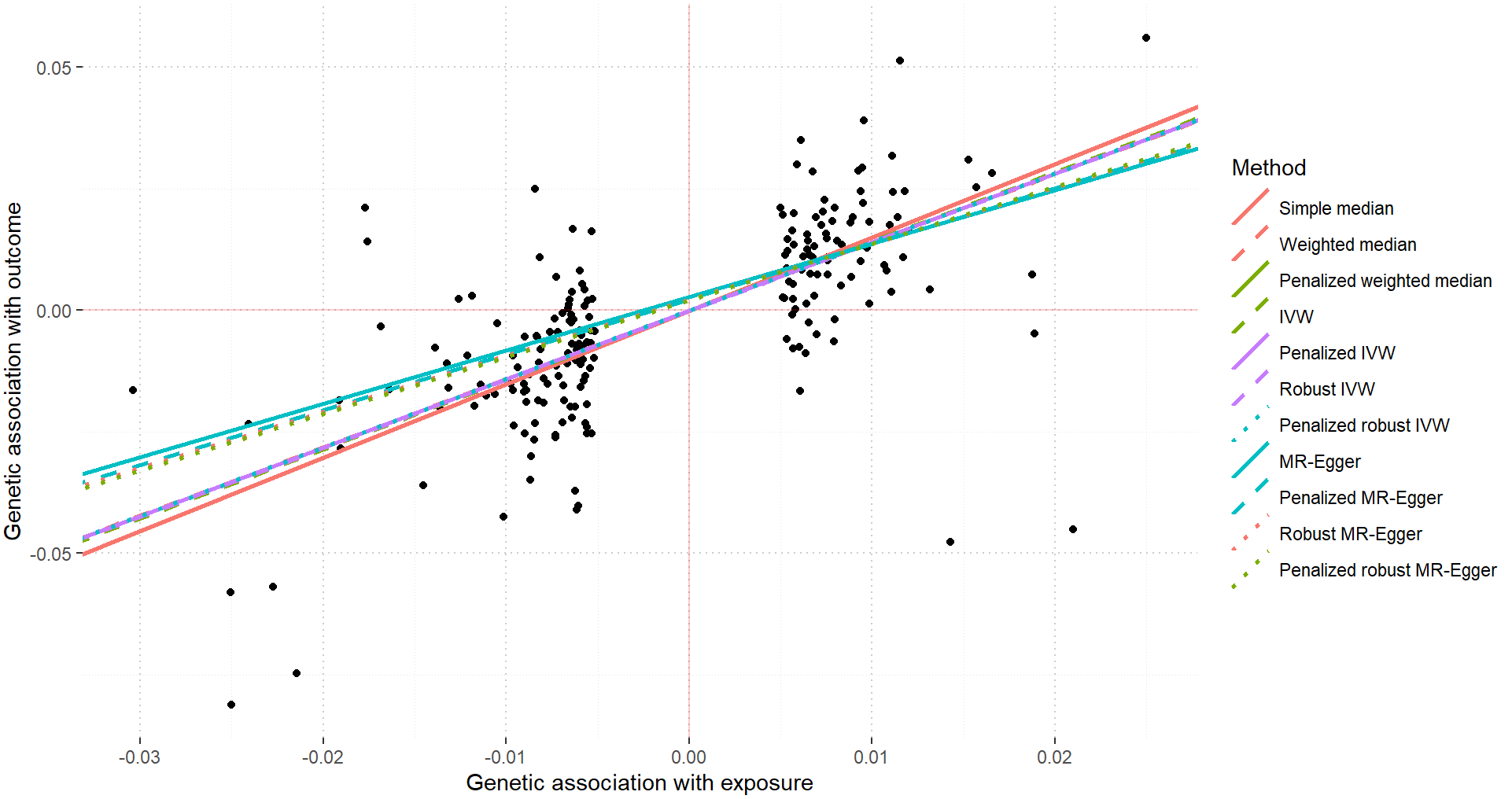


| Methods | OR | CI 95% | | P-value |
| --- | --- | --- | --- | --- |
| Simple median | 4.57 | 3.56 | 5.92 | <0.001 |
| Weighted median | 3.86 | 3.01 | 4.97 | <0.001 |
| Penalized weighted median | 4.14 | 3.18 | 5.36 | <0.001 |
| Inverse variance weighting (IVW) | 4.18 | 3.40 | 5.13 | <0.001 |
| Penalized IVW | 4.14 | 3.45 | 4.90 | <0.001 |
| Robust IVW | 4.14 | 3.40 | 5.05 | <0.001 |
| Penalized robust IVW | 4.14 | 3.42 | 4.95 | <0.001 |
| MR-Egger | 2.98 | 1.64 | 5.40 | <0.001 |
| Penalized MR-Egger | 3.09 | 1.88 | 5.10 | <0.001 |
| Robust MR-Egger | 3.13 | 1.68 | 5.87 | <0.001 |
| Penalized robust MR-Egger | 3.19 | 1.75 | 5.75 | <0.001 |


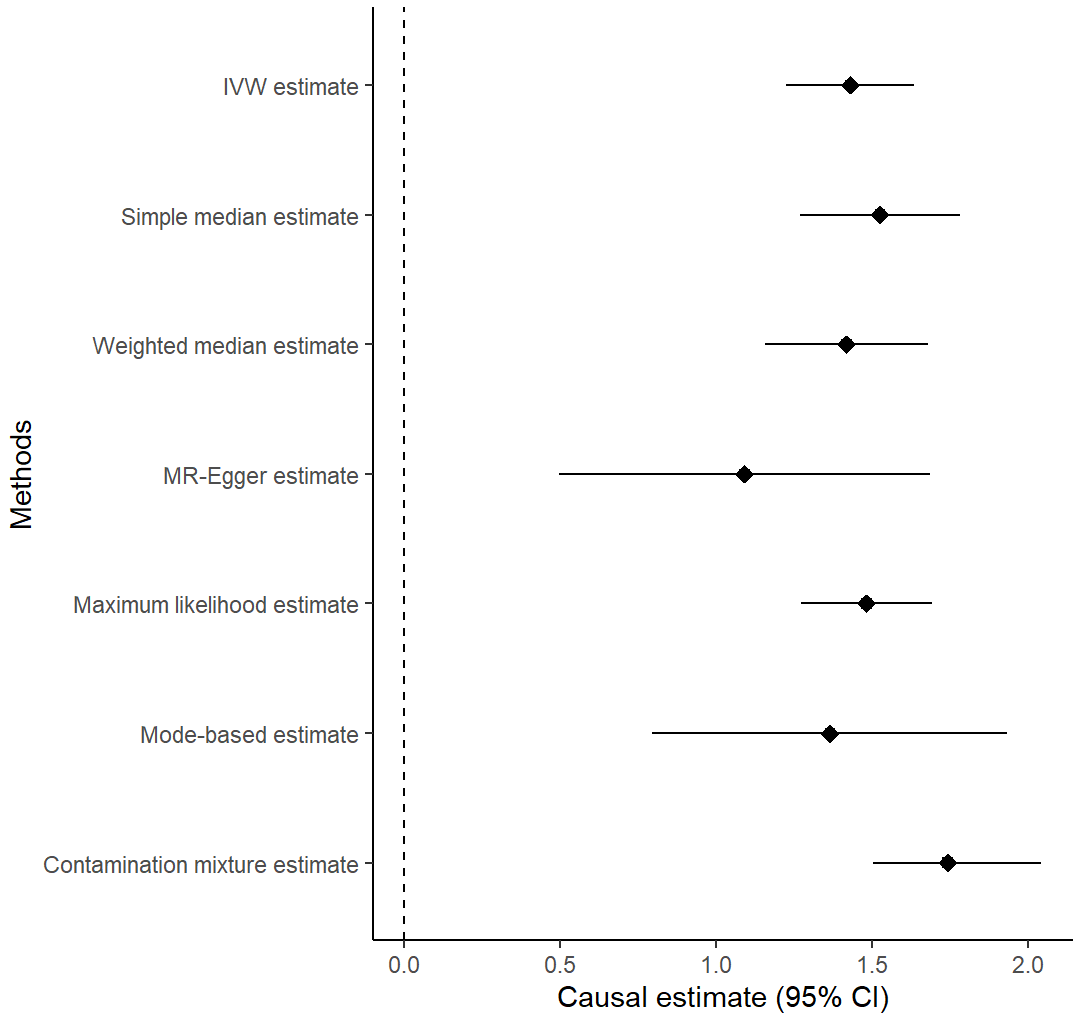


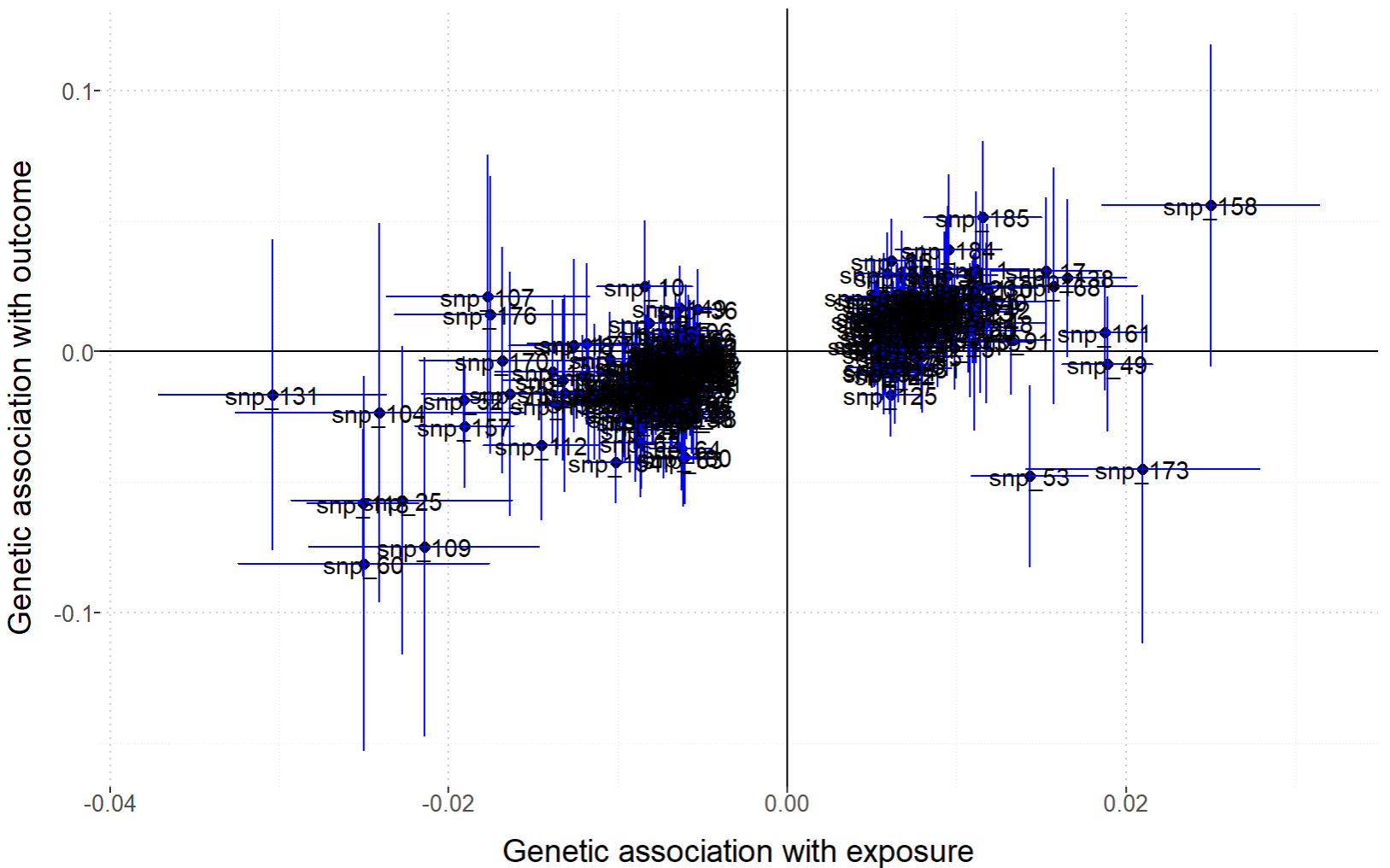


**Figure S5: Leave-one-out plot to assess if a single variant is driving the association between C-reactive protein and heart failure.**


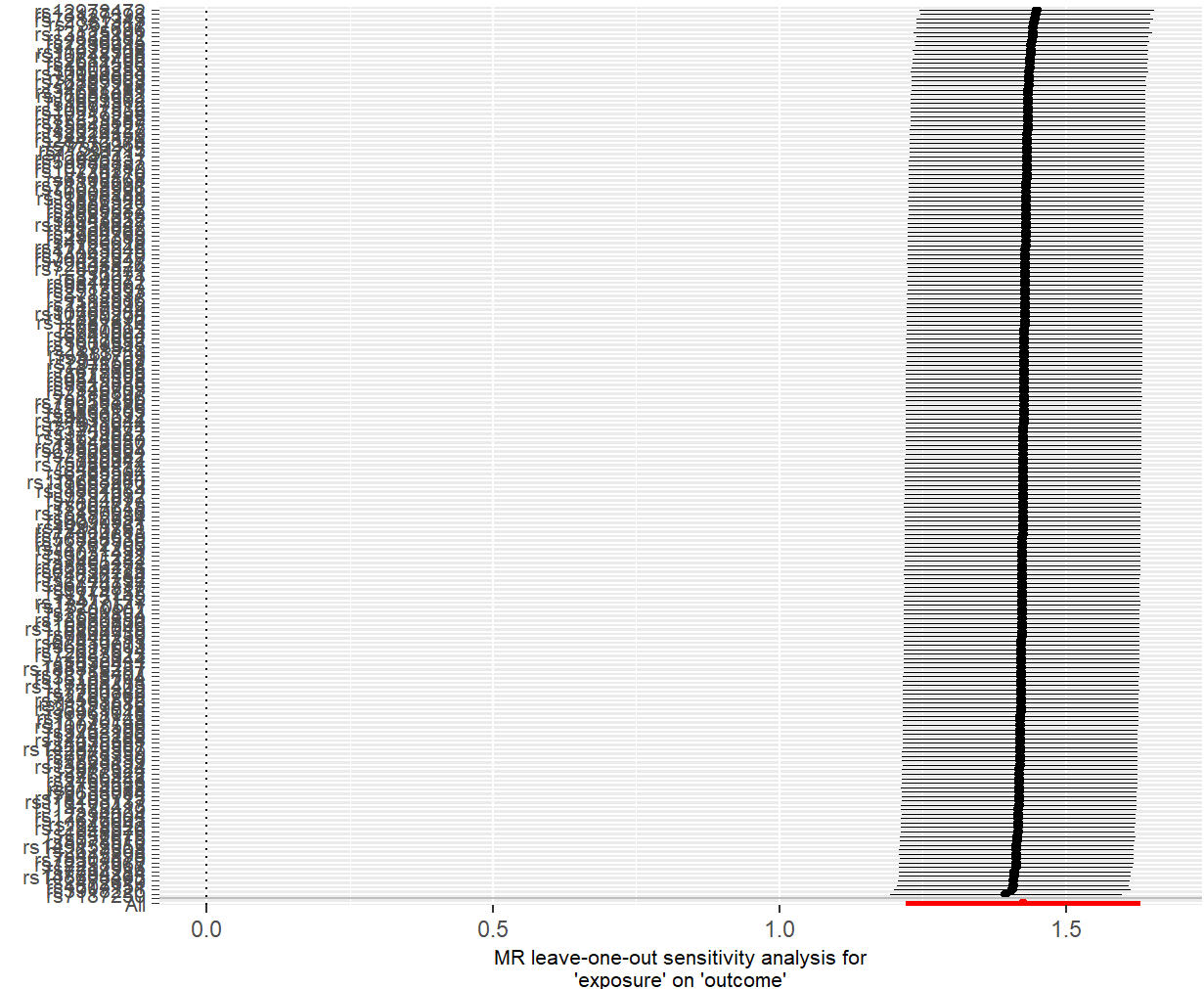


**Figure S6: Forest plot of variant specific inverse variance estimates for causal association between c-reactive protein and heart failure**


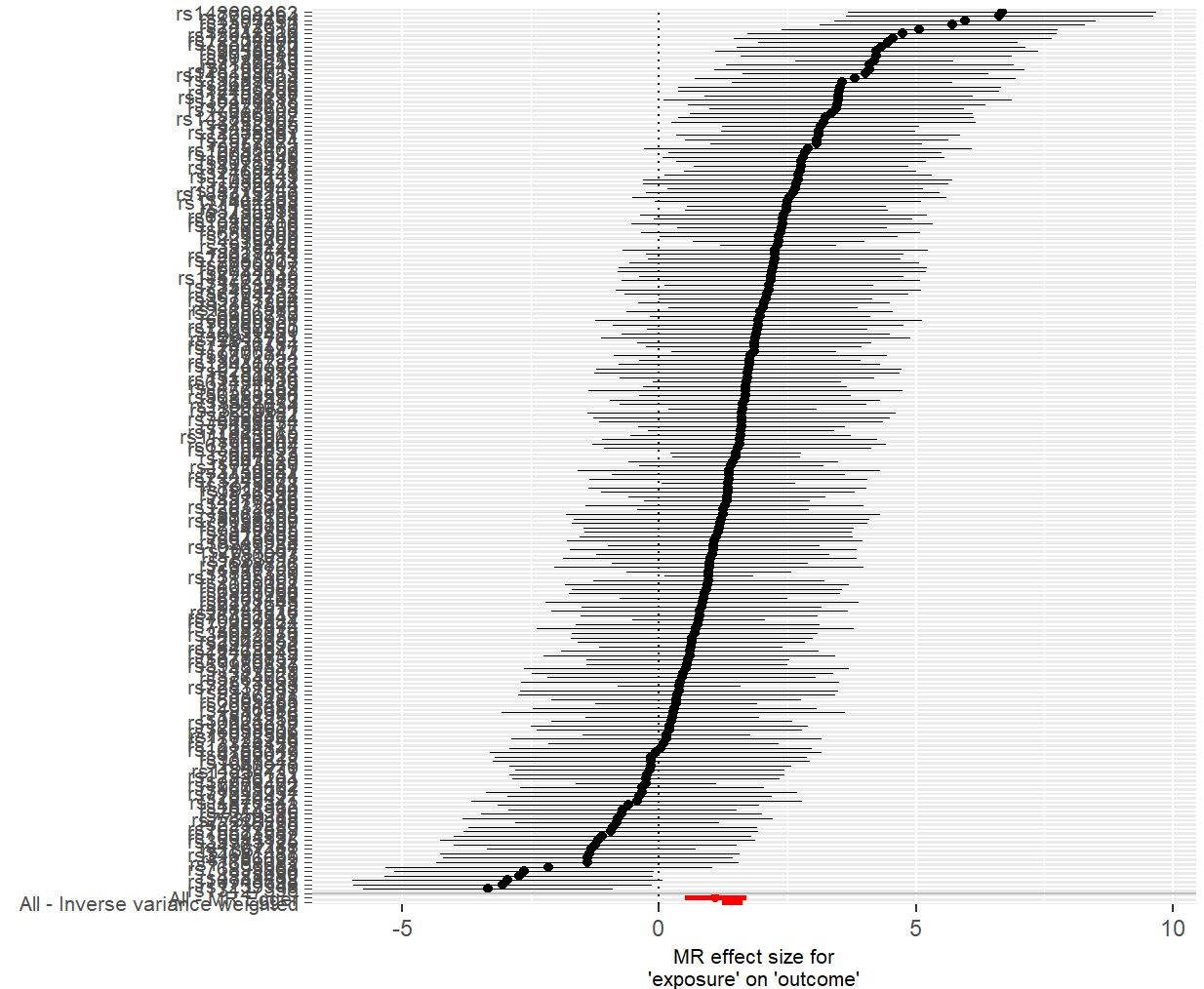


**Figure S7. Funnel plot of causal association between c-reactive protein and heart failure**


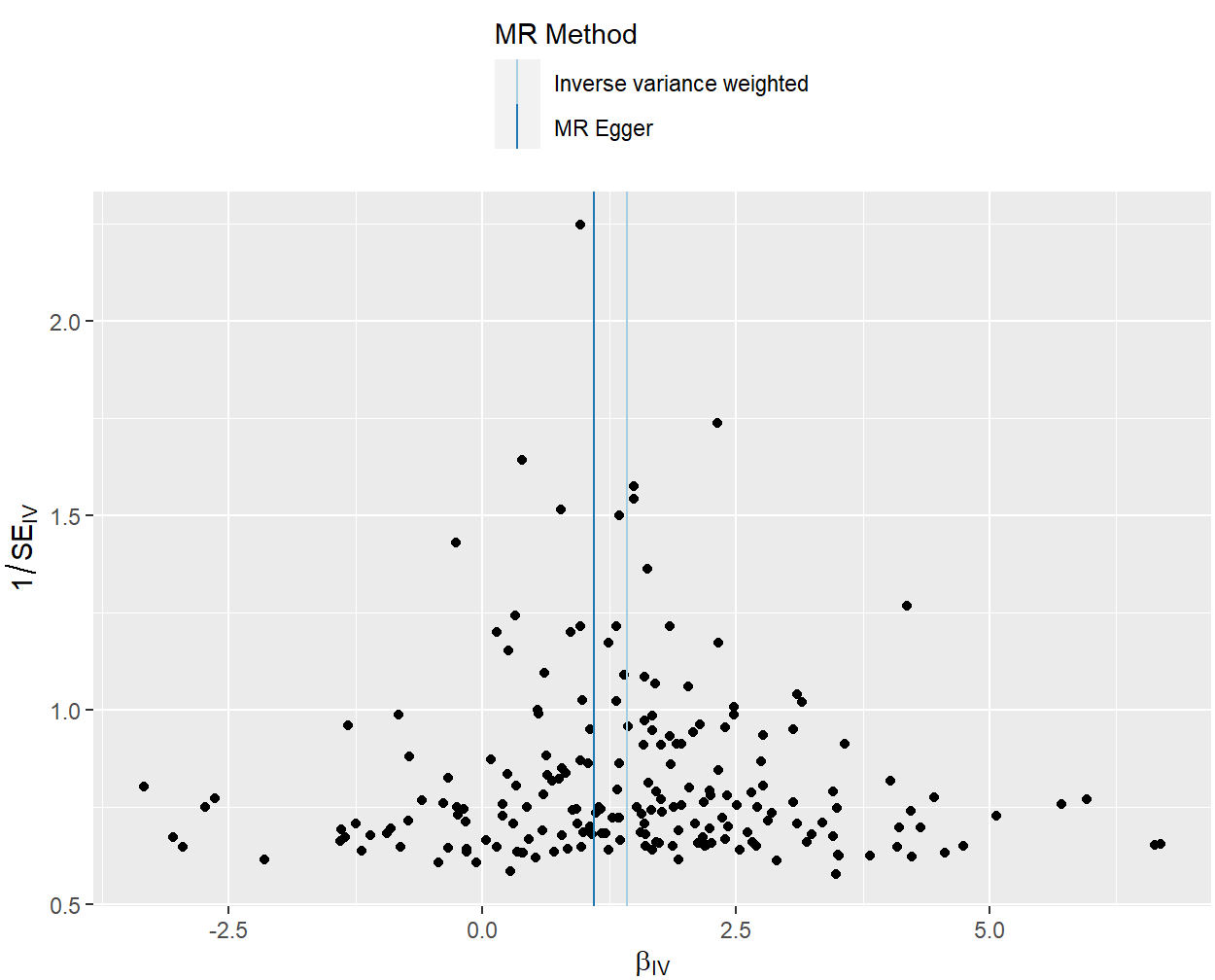


**Figure S8. Causal association between heart failure and c-reactive protein**


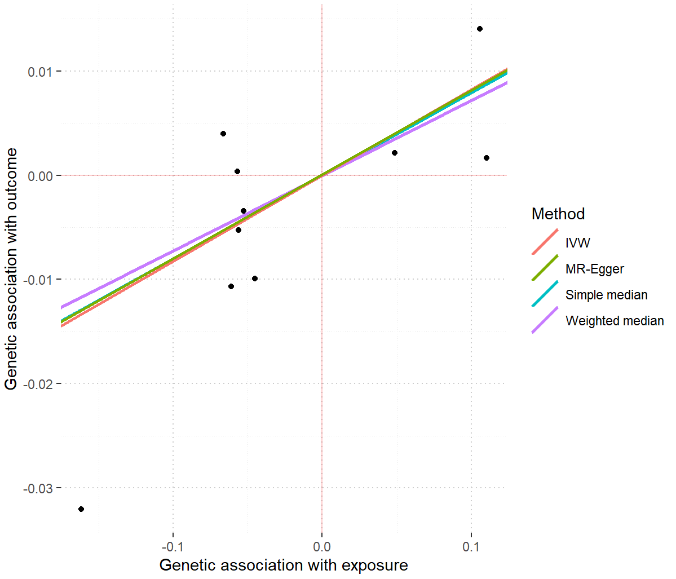

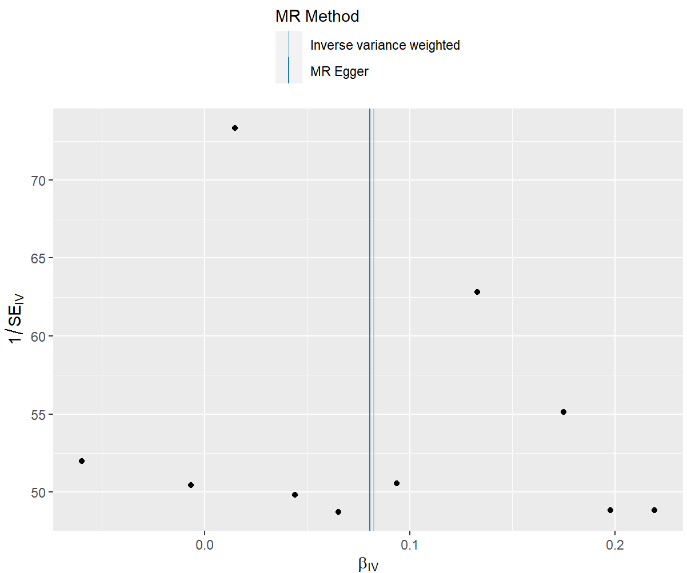

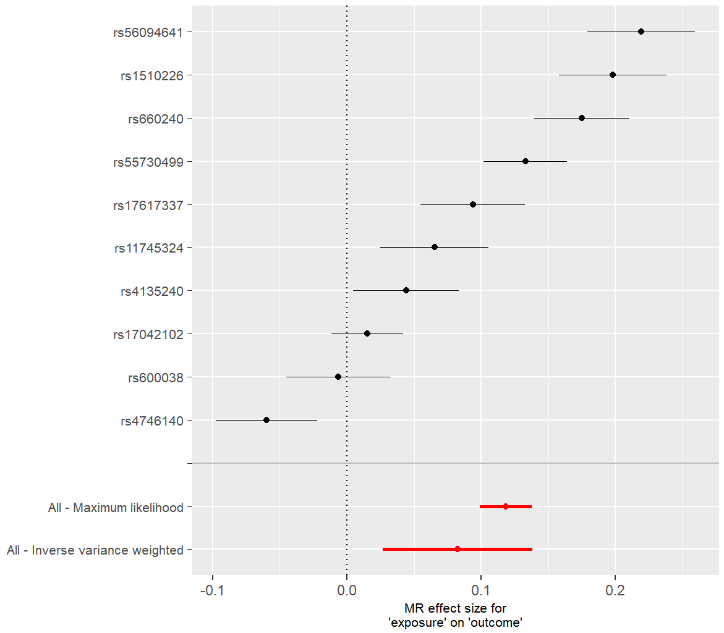

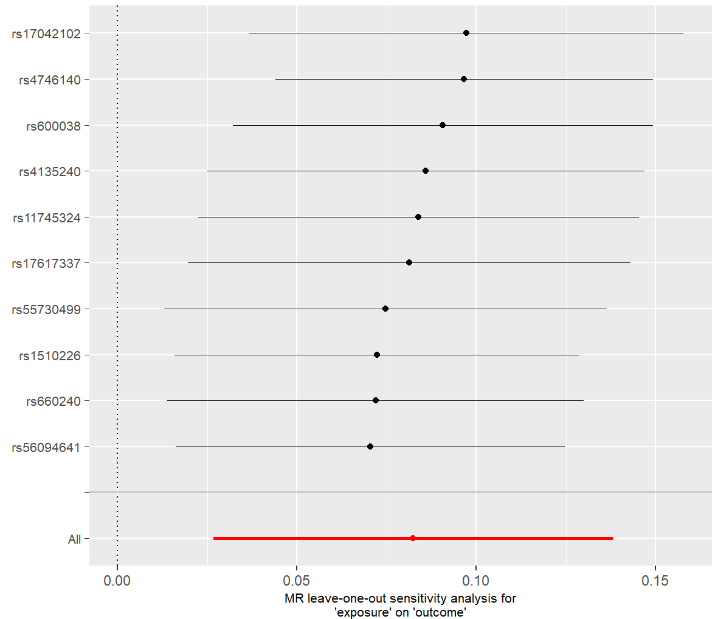

Supplement: Supplementary file 2 — Additional File 2: Description of supplementary materials [file 12872_2023_3149_MOESM2_ESM.docx]
